# Integrated Analysis and Finding Reveal Anti–Liver Cancer Targets and Mechanisms of Pachyman (*Poria cocos* Polysaccharides)

**DOI:** 10.3389/fphar.2021.742349

**Published:** 2021-09-17

**Authors:** Li Qin, Dongning Huang, Jian Huang, Fuhui Qin, Haixin Huang

**Affiliations:** Department of Oncology, Liuzhou Worker's Hospital, Liuzhou, China

**Keywords:** liver cancer, Pachyman, network pharmacology, molecular docking, biomarkers 3

## Abstract

This bioinformatics study aimed to characterize and certify crucial anti-cancer targets, functional processes, and molecular mechanisms of Pachyman in treating hepatocellular carcinoma (HCC) by using pharmacology network and molecular docking analyses, by experimental validation. The crucial anti-HCC targets of Pachyman, including ALB, VEGFA, TNF, CASP3, SRC, EGF, CXCR4, STAT3, HRAS, HSP90AA1, MMP9, BCL2L1, FGF2, and PTPRC, were identified. In addition, the correlative networks of all crucial biotargets of Pachyman in treating HCC were created accordingly. Functionally, these crucial genes were correlated using angiogenesis and neoplastic metastasis of HCC. Interestingly, the molecular docking findings indicated that ALB and VEGFA in HCC might be potent pharmacological targets of Pachyman. In experimental validation, the clinical samples of HCC showed reduced ALB protein expression and increased VEGFA protein level. Following Pachyman treatments *in vitro*, the intracellular level of ALB protein was elevated, whereas the cellular content of VEGFA protein was downregulated. Taken together, current bioinformatics findings based on pharmacology network and molecular docking analyses elucidate the detailed molecular targets and signaling mechanisms of Pachyman in treating HCC. Interestingly, validated biotargets of ALB and VEGFA may be main potential biomarkers for detecting HCC medically.

## Introduction

Hepatocellular carcinoma (HCC) refers to a malignant tumor, occurring in liver tissue *in situ*. Statistically, HCC cases account for a majority of cancer-causing deaths worldwide (McGlynn et al., 2021). As revealed in epidemiological assay, HCC is one of the deadly tumors in China, featured with elevated morbidity and mortality yearly (Fu and Wang, 2018). Notably, early symptoms of HCC may be inconspicuous during clinical characterization before the tumor develops as malignant and invasive. Accordingly, most of the HCC patients are diagnosed with advanced stages during initial examination, eventually leading to high mortality and undesired metastasis (Llovet et al., 2018; Llovet et al., 2021). For precision medicine, it is particularly necessary to identify more candidate anti-HCC targets for effective diagnosis, prognosis, and treatment (Anwanwan et al., 2020). In addition, some naturally occurring ingredients may be used for HCC treatment. Pachyman, *Poria cocos* polysaccharides, is a biologically active component that is isolated from the *Poria cocos* plant (Sun, 2014). Beneficially, Pachyman has been proven to exert potent pharmacological properties, such as antioxidation, anti-tumor action, hepatoprotection, enhancing immunity, and eliminating free radicals (Wu et al., 2018; Li et al., 2019; Liu et al., 2020). Although well-evidenced anti-cancer benefits have been found, there are still only few studies regarding the pharmacological effects of Pachyman in treating HCC. Intriguingly, an attractive strategy using network pharmacology and molecular docking analyses can function as an emerging and promising tool for revealing detailed biotargets and molecular mechanisms of candidate natural agents for treating medical diseases (Li et al., 2020a; Li et al., 2021a), including liver disease (Su et al., 2019), coronavirus disease 2019 (COVID-19) (Qin et al., 2021), and meningitis (Li et al., 2021b). Therefore, the present study was designed to optimize network pharmacology and molecular docking methods in identifying the bioinformatics findings of Pachyman in treating HCC, including detailed biological targets, functional processes, and signaling pathways. Experimentally, some bioinformatics data were validated for potential clinical use of Pachyman in treating HCC in future.

## Materials and Methods

### Screening of Anti-HCC Candidate Targets of Pachyman

All pharmacological targets of Pachyman were collected from databases of PharmMapper and Swiss Target Prediction databases. In addition, HCC-related targets were obtained from databases of OncoDB.HCC and Liverome. Furthermore, these identifiable targets of Pachyman and HCC were determined by using the FunRich software to construct an intersection map of Pachyman in treating HCC, as described by Li et al. (2021c).

### Construction of an Integrated Network in Anti-HCC Targets of Pachyman

The available anti-HCC targets of Pachyman were imported to the STRING database for presenting protein–protein interaction (PPI) data with the confidence score greater than 0.9, and then introduced to the Cytoscape software for constructing a PPI map of Pachyman against HCC targets. The PPI network was modeled as a visualized graph, in which the nodes represented genes and the edges indicated the interaction proteins encoded by the related genes (Li et al., 2021d).

### Identification and Construction Hub Network of Crucial Targets

Additionally, Network Analyzer in Cytoscape tool was used to determine topological parameters, such as average degree and maximum degree of PPI network of Pachyman in treating HCC, and then the crucial targets were screened and identified according to the contrivable degree value of the targets. Moreover, these crucial targets with interaction network were featured and visualized by using a software platform of Cytoscape for integrating complex networks, as described by Li et al. (2021e).

### Enrichment and Function Analyses of Crucial Targets

Subsequently, Gene Ontology (GO) function and Kyoto Encyclopedia of Genes and Genomes (KEGG) pathway enrichment assays were generated by using the Metascape online platform. Additionally, the biological functions and pathway enrichment findings of all crucial targets were featured and visualized by using the OmicShare cloud platform before generating advanced bubble diagrams according to their *p*-values. Furthermore, the crucial target-signaling pathway network of Pachyman in treating HCC was constructed by using the Cytoscape software (Li et al., 2020b).

### Molecular Docking Analysis

The molecular structure of the Pachyman compound was obtained from the PubChem database. The crucial target/protein structure in HCC was obtained from the Protein Data Bank database. Magnetometric minimum force field was obtained by using the three-dimensional structure of ChemBio3D Draw module in the ChemBioOffice software (version 2010). The PDBQT structure file necessary for virtual screening was created through the Raccoon software, and the docking active center was defined through the grid box function setting in the software. Data reliability was determined according to the size of the root-mean-square deviation of the docked and the original ligand molecules. Generally, it was designed in a way that the root-mean-square deviation that is ≤4 was the threshold for conforming the docked ligand to match the related original ligand (Nong et al., 2020).

### Clinical Study

In a human study, 10 patients with HCC were detected, and clinical samples were collected for experimentative verification. All patients were medically diagnosed for HCC through clinical images and histological staining examination. HCC and HCC-free samples were harvested through surgical operation, followed by immunostaining and analyzing. These human protocols were approved by the Liuzhou Worker’s Hospital ethics committee, and correlative experiments were conducted based on the principles of the Declaration of Helsinki (Li et al., 2020c; Li et al., 2021f).

### Cell Culture Study

A human liver cancer cell line of HepG2 was treated with Pachyman (Shanghai Yuanye Biotechnology Co., Ltd. China) at different doses of 0, 25, and 50 μM for 48 h. The cell proliferation was determined by using the commercially available reagents of Cell Counting Kit 8. In addition, the protein expressions of ALB and VEGFA were measured by using enzyme-linked immunosorbent assay (ELISA) kits. More experimental procedures have been described in previous reports (Wu et al., 2017; Wu et al., 2019).

### Statistical Analyses

The statistical results were indicated as mean ± standard deviations (SD). Statistical assay was performed by using the Statistical Product and Service Solutions 19.0 software (IBM Corporation, Chicago, IL, United States). Two different comparison groups were determined through one-way analysis of variance, followed by Tukey's post hoc test. The statistical significance was set as *p* < 0.05.

## Results

### Potential Targets and Establishment of the PPI Network

**FIGURE 1:**
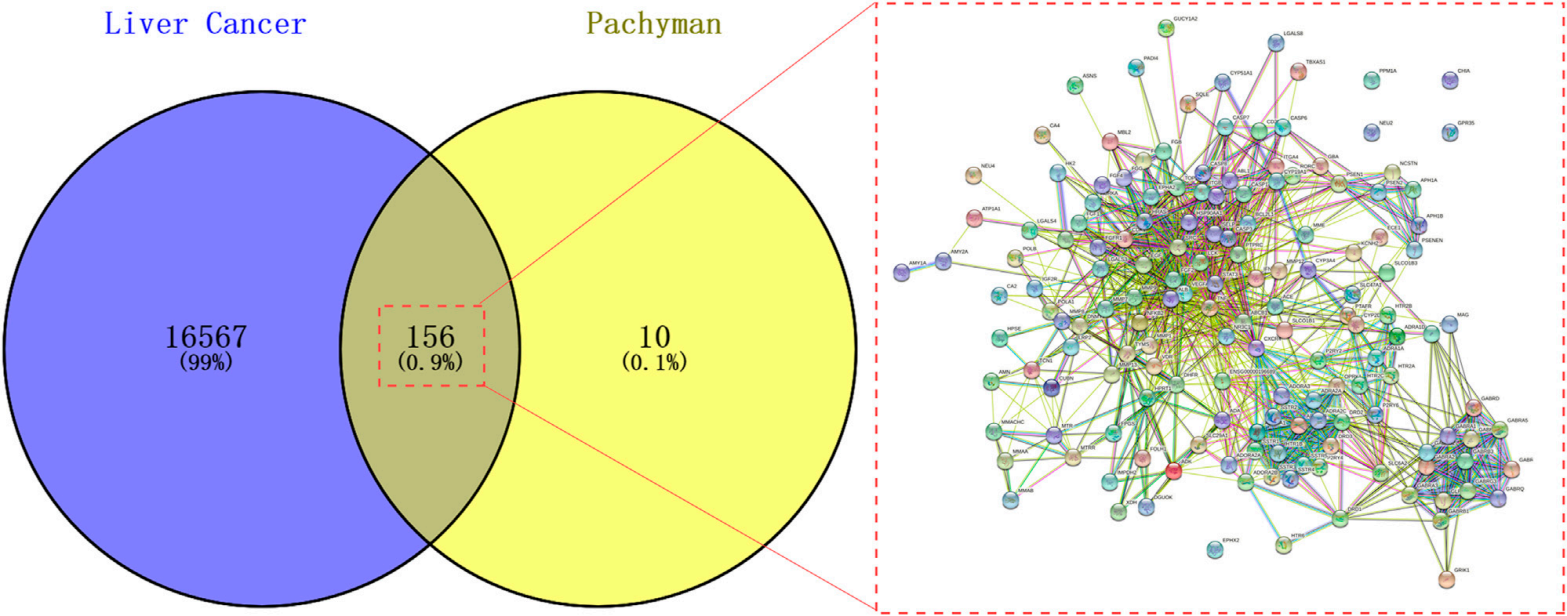
Candidate targets of a PPI network of Pachyman in treating HCC for identifying pharmacological and pathological biotargets.

As shown in the Venn diagram (Figure 1), the data reported a total of 16,763 HCC-associated genes and 166 Pachyman-associated genes. As a result, 156 intersection genes of Pachyman and HCC were obtained when all duplicate genes were excluded. Visibly, the PPI network of the intersection genes showed 57 nodes and 139 edges (Figure 1).

### Identification of Crucial Targets in Anti-HCC Targets of Pachyman

**FIGURE 2:**
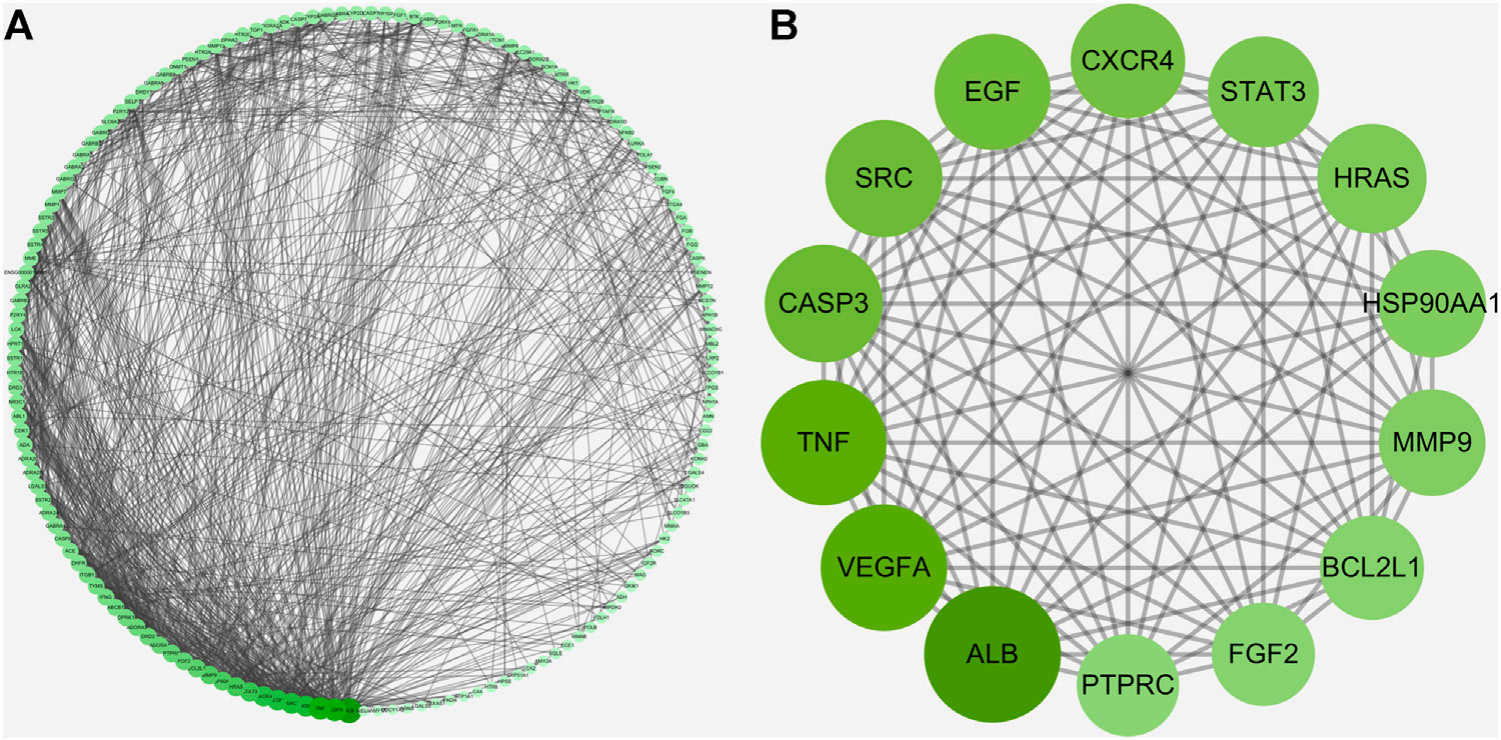
Hub targets of a PPI network **(A)** for uncovering all crucial targets of Pachyman in treating HCC **(B)**.

The topological data indicated a close relevance among the intersection genes, characterized in the PPI network map (Figure 2A). As revealed in Figure 2B, the top 14 crucial targets of ALB, VEGFA, TNF, CASP3, SRC, EGF, CXCR4, STAT3, HRAS, HSP90AA1, MMP9, BCL2L1, FGF2, and PTPRC were identified accordingly.

### GO and KEGG Enrichment Findings of Crucial Targets

**FIGURE 3:**
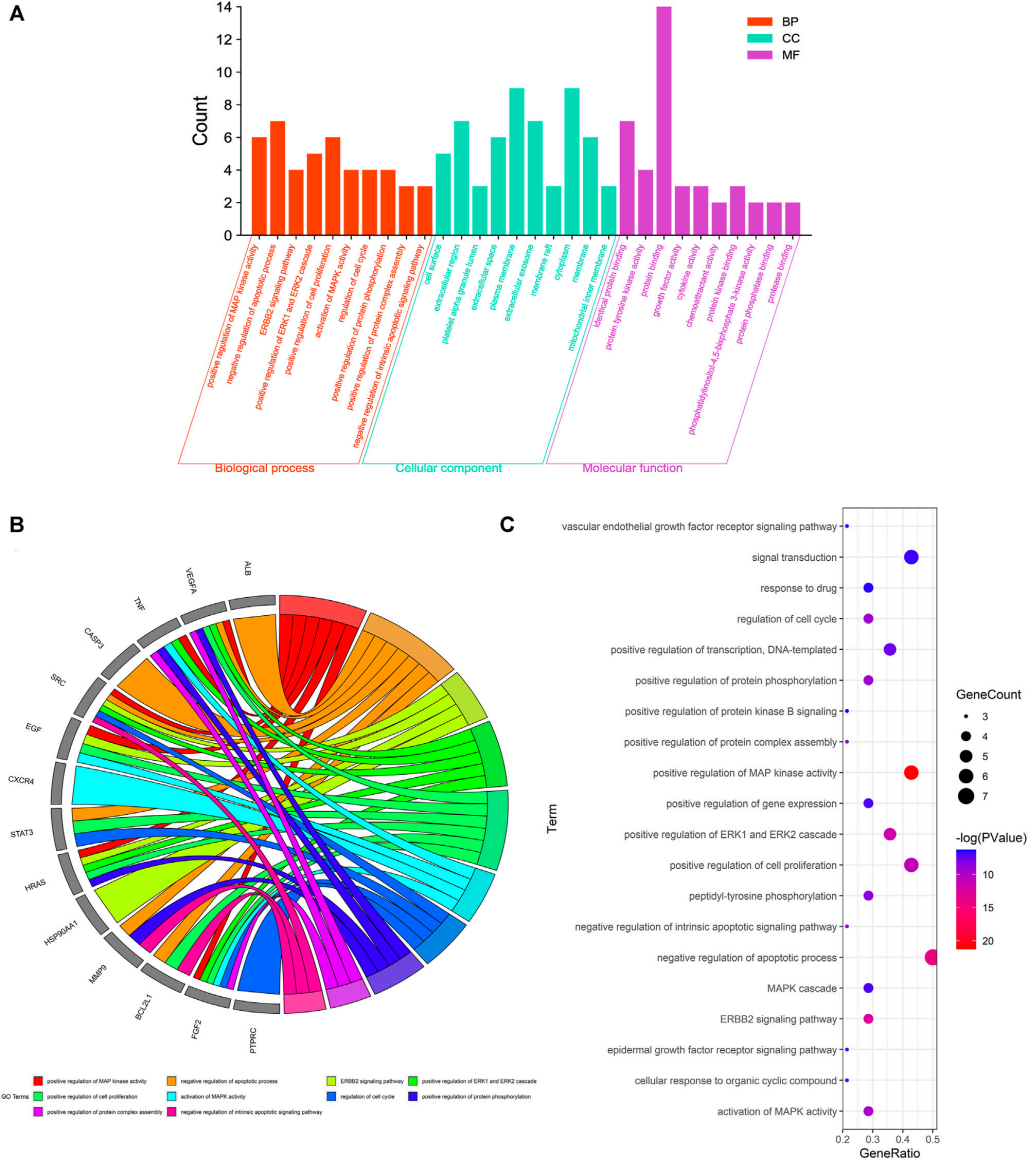
GO-based enrichment analysis for disclosing the top 20 biological processes of Pachyman in treating HCC, as shown in bar chart **(A)**, circle diagram **(B)**, bubble graph **(C)**.

**FIGURE 4:**
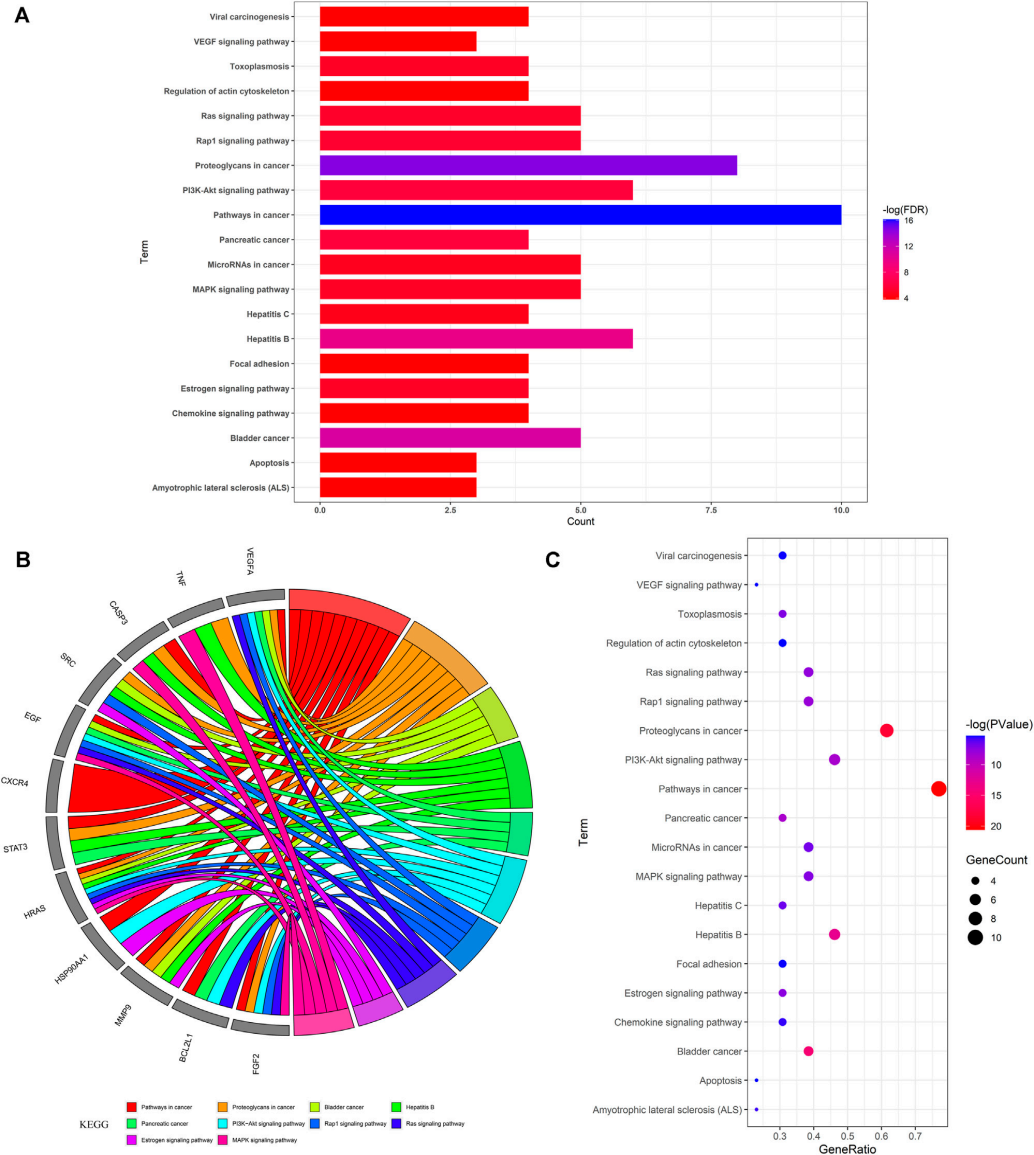
KEGG-based enrichment analysis for revealing the top 20 molecular pathways of Pachyman in treating HCC as shown in bar chart **(A)**, circle diagram **(B)**, bubble graph **(C)**.

**FIGURE 5:**
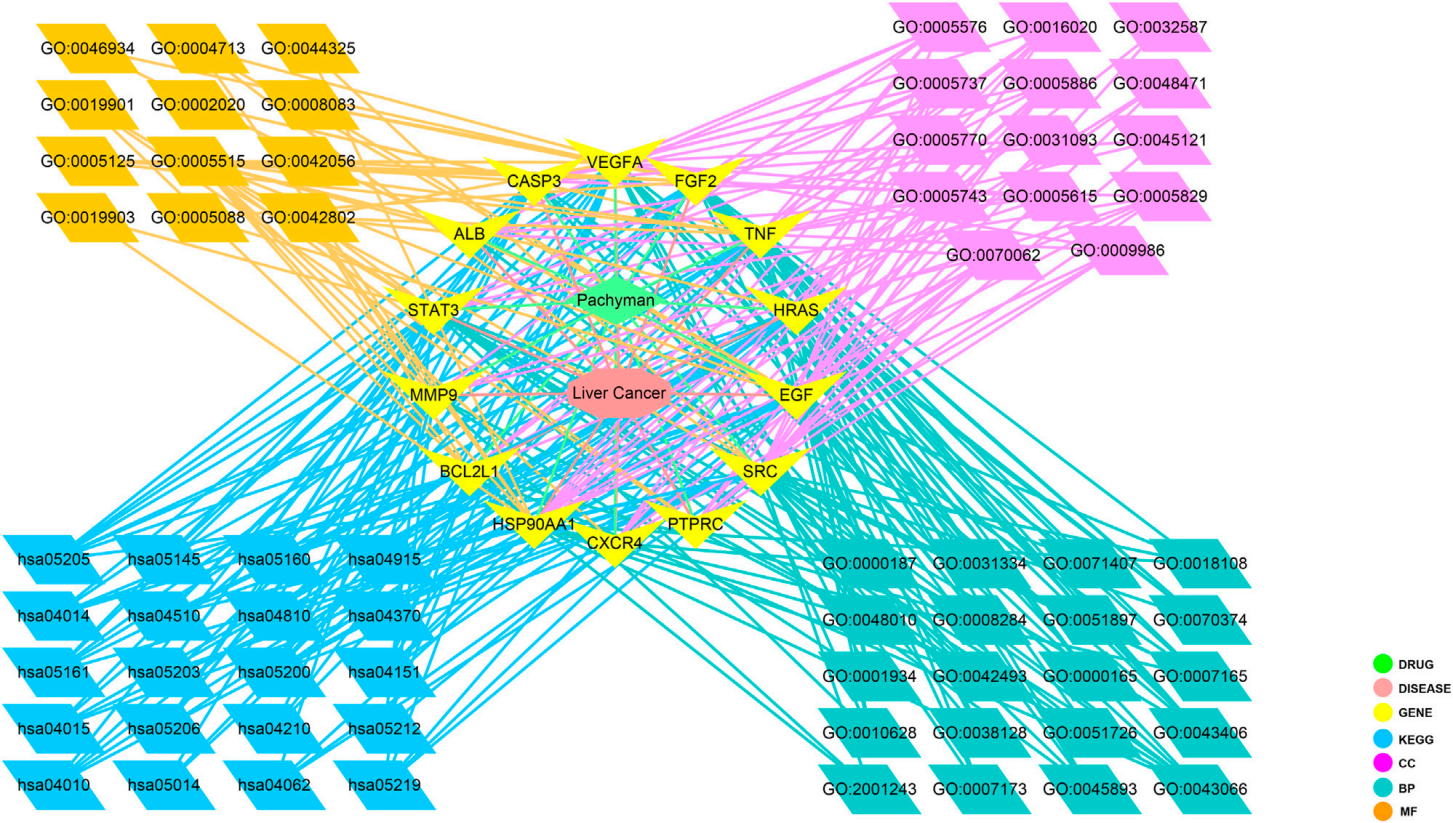
An integrative map of disease and ingredient-target-signaling pathway network of Pachyman in treating HCC.

The biological functional processes and molecular pathways of the crucial targets in Pachyman for treating HCC were enriched and characterized respectively. The top 20 biological processes of Pachyman in treating HCC are illustrated in Figure 3A–C. The anti-cancer effects of Pachyman in treating HCC were mainly related to the regulation of the following: positive regulation of MAP kinase activity, negative regulation of apoptotic process, ERBB2 signaling pathway, positive regulation of extracellular signal-regulated kinase (ERK)—ERK1 and ERK2—cascades, positive regulation of cell proliferation, activation of mitogen-activated protein kinase (MAPK) activity, regulation of the cell cycle, positive regulation of protein phosphorylation, positive regulation of the protein complex assembly, negative regulation of the intrinsic apoptotic signaling pathway, peptidyl-tyrosine phosphorylation, positive regulation of DNA-templated transcription, regulation of the epidermal growth factor receptor signaling pathway, regulation of cellular response to organic cyclic compound, MAPK cascade, positive regulation of gene expression, signal transduction, vascular endothelial growth factor receptor signaling pathway, response to drug, and positive regulation of protein kinase B signaling. As is revealed in pharmacological mechanisms, the KEGG pathways of Pachyman in treating HCC included the following: pathways in cancer, proteoglycans in cancer, bladder cancer, hepatitis B, pancreatic cancer, PI3K-Akt signaling pathway, Rap1 signaling pathway, Ras signaling pathway, estrogen signaling pathway, MAPK signaling pathway, toxoplasmosis, microRNAs in cancer, hepatitis C, amyotrophic lateral sclerosis (ALS), chemokine signaling pathway, viral carcinogenesis, focal adhesion, VEGF signaling pathway, apoptosis, and regulation of actin cytoskeleton (Figures 4A–C). Collectively, the integrated network diagram with detailed Pachyman–anti-HCC findings can be visualized in Figure 5.

### Molecular Docking Findings

**FIGURE 6:**
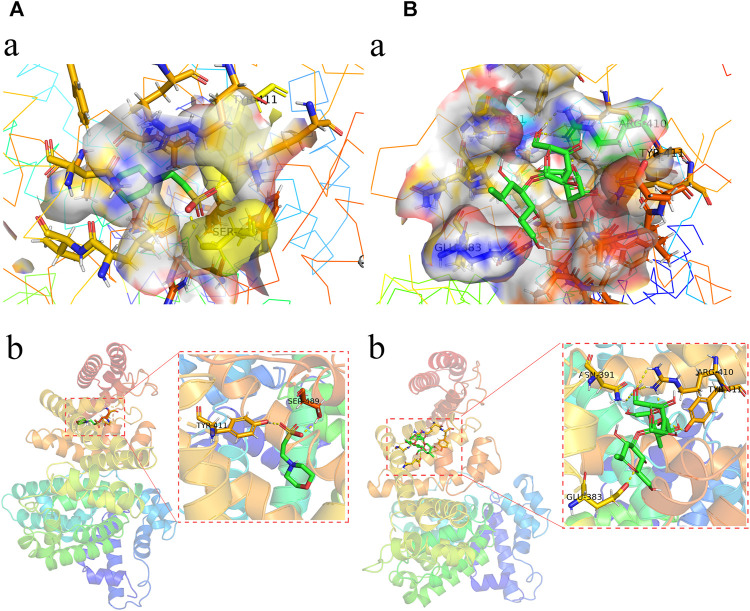
The molecular docking analysis shows potent binding activities of Pachyman with 6QIO in ALB as a crucial target in HCC.

**FIGURE 7:**
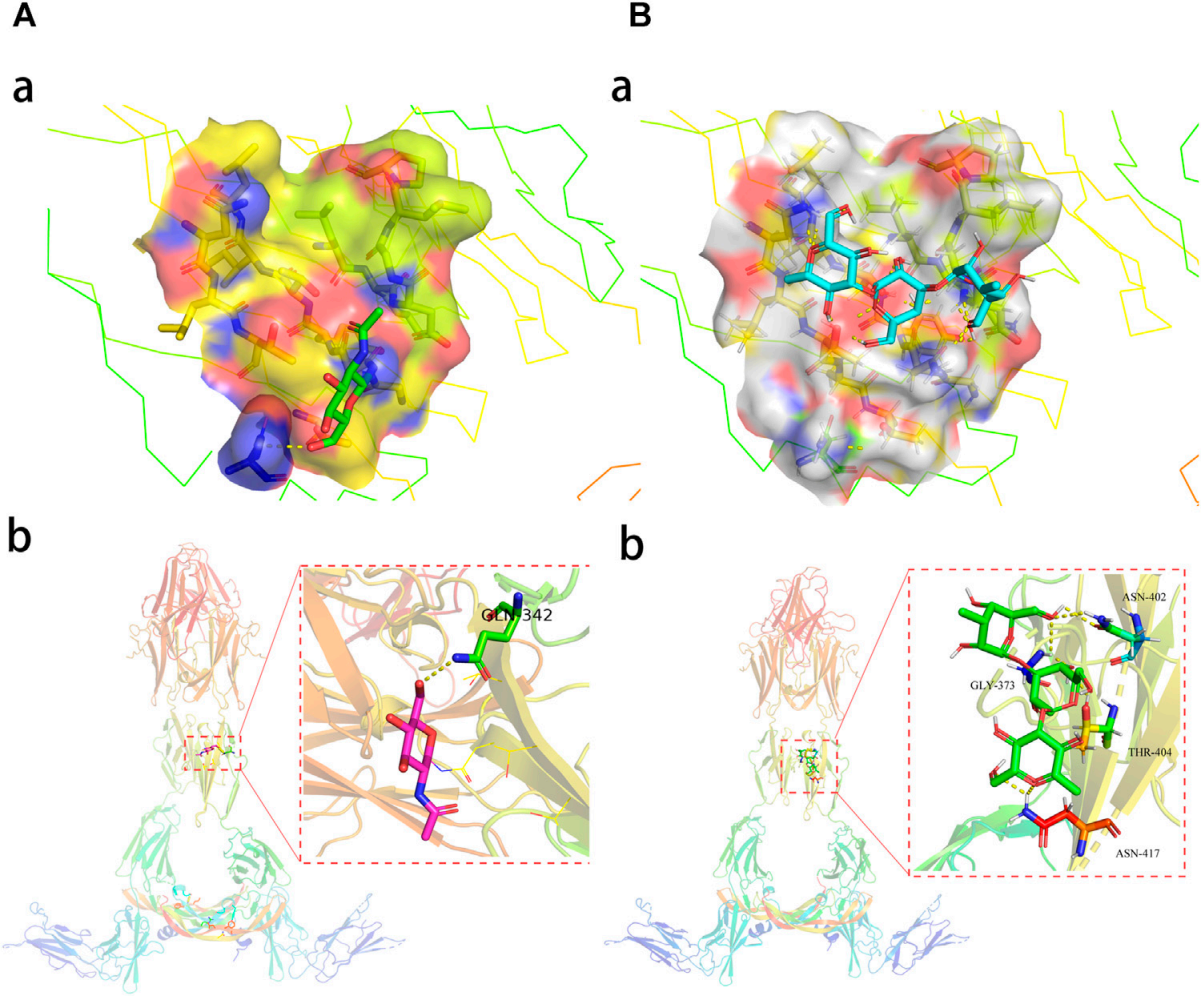
The molecular docking data–suggested potent binding activities of Pachyman with 5T89 in VEGFA as a crucial target in HCC.

As shown in the ALB protein (Figure 6), the root-mean-square deviation of the original ligand is 2.713 Å. The hydrogen bonding of the pro-ligand MES to 6QIO protein acted on the amino acid residues TYR-411 (1.6Å) and SER-489 (2.0 Å). Pachyman formed bonds with the amino acid residues of TYR-411 (1.7 Å), ASN-391 (2.6 Å), ARG-410 (2.6 Å), and GLU-383 (2.2 Å) (Figure 6). As shown for VEGFA protein, the root-mean-square deviation of the original ligand was 2.586 Å. The hydrogen bonding of the pro-ligand NAG and 5T89 protein acted on the amino acid residues of GLN-342 (3.2 Å). Pachyman formed bonds with amino acid residues of ASN-402 (2.6 Å), THR-404 (2.5 Å), GLY-373 (2.8 Å), and ASN-417 (2.6 Å) (Figure 7).

### Validated Data in Clinical HCC Samples

**FIGURE 8:**
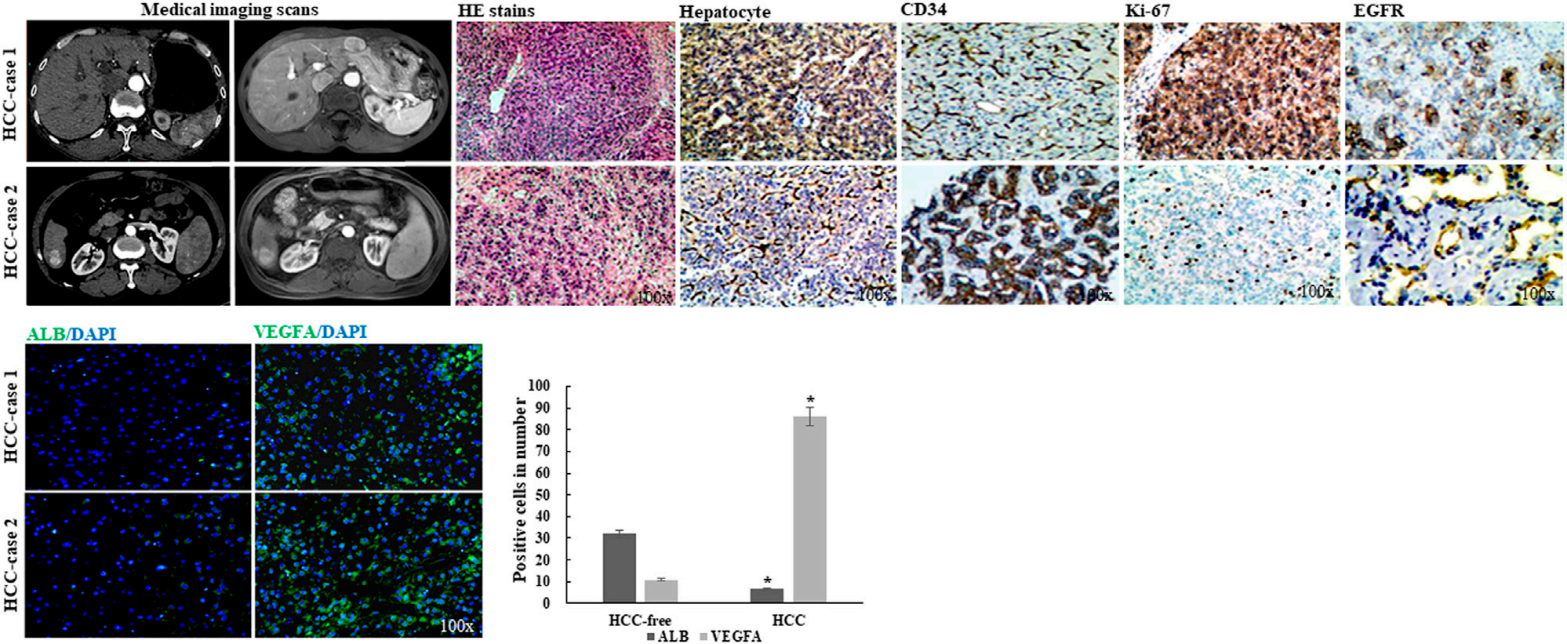
Characterized imaging and immunostaining findings of clinical HCC samples, and validated data based on molecular docking analysis.

To further validate the molecular docking findings, the HCC and HCC-free clinical samples were collected for experimental tests. The HCC cases were well characterized medically with medical imaging scans and pathological diagnosis (hematoxylin and eosin stain (HE) and positive cells for hepatocyte, CD34, Ki-67, and EGFR expressions). Additionally, the data from the fluorescence immunostaining assay showed that the HCC samples resulted in significant downregulation of ALB expression and elevation of VEGFA expression when compared with those in HCC-free controls (*p* < 0.05) (Figure 8).

### Validated Data in Pachyman-Treated HCC Cells

**FIGURE 9:**
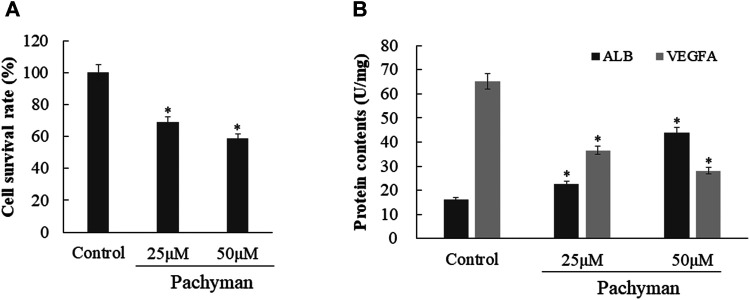
Validated data *in vitro* with Pachyman-treated liver cancer cells according to molecular docking findings, as shown in cell proliferation test **(A)**, target protein determination **(B)**.

The experimental data *in vitro* suggested that Pachyman-treated HepG2 cells showed markedly reduced cell proliferation in comparison with the non-treated control (*p* < 0.05) (Figure 9A). In the quantitative test performed by using ELISA, the Pachyman-treated HepG2 cells exhibited increased ALB expression (*p* < 0.05) and reduced VEGFA content (*p* < 0.05) (Figure 9B).

## Discussion

In the current bioinformatics findings of this study, network pharmacology–based analysis had identified individual and crucial targets, biological processes, and signaling pathways of Pachyman in treating HCC. The anti-HCC actions of Pachyman might be related to inducing cell death, enhancing immunologic function, and meliorating tumor microenvironmental regulation, as has been revealed in the GO-based analysis. Additionally, anti-proliferation pathways (such as apoptosis, and PI3K-Akt and MAPK signaling pathways), anti-invasiveness pathway (such as Ras and Rap1 signaling pathway), anti-angiogenesis pathway (such as VEGF signaling pathway), and other pathways (such as MicroRNAs in cancer) were all identified in Pachyman treating HCC actions, as revealed in the KEGG-based findings. During further screening of crucial targets by using molecular docking analysis, the Pachyman-activated anti-HCC core targets, including ALB and VEGFA, were identified accordingly, characterized with potent binding activities of Pachyman with 6QIO and 5T89 proteins in HCC. ALB, a monomeric protein, plays principally as a carrier protein for fatty acids, steroids, and thyroid hormones (Garcia-Martinez et al., 2013). Medically, serum ALB may be used for evaluating the nutritional condition in cancer patients, as malnutrition can be a function of prognostic significance in cancer cases (Gupta and Lis, 2010). It has been reported by using *in situ* hybridization test that endogenous albumin mRNA may serve as a sensitive marker for detecting primary hepatic carcinoma (Chen et al., 2021). In addition, the prognostic value of the albumin score in HCC cases has been identified clinically (Feng et al., 2020). VEGFA, a glycosylated mitogen, can induce vascular permeability, vasculogenesis, and endothelial cell proliferation or growth (Ballmer-Hofer, 2018). In tumor-related angiogenesis, VEGFA-based response mediates numerous functional effectors, including phosphoinositide 3 kinase (PI3K)/Akt, p38 MAPK, and ERKs (Claesson-Welsh and Welsh, 2013). In addition, VEGFA-induced HCC development and pathogenesis may be a potential pharmacological target for treating hepatocellular carcinoma (Morse et al., 2019). In the present validated experiments, clinical HCC samples showed decreased ALB expression and elevated VEGFA content. As shown in *in vitro* Pachyman treatments, intracellular protein of ALB was upregulated and VEGFA protein expression was reduced. These validated findings demonstrated that ALB and VEGFA may be the anti-HCC pharmacological targets activated by Pachyman. The limitation of the present study is that further *in vivo* experimental validation is needed for potential use of Pachyman in clinical application.

## Conclusion

Collectively, the bioinformatics and validation findings revealed the pharmacological targets and mechanisms of Pachyman in treating HCC. Specially, computational identification of ALB and VEGFA proteins/genes may functionally serve as potential biomarkers in screening for and treating HCC.

## Data Availability

The original contributions presented in the study are included in the article/Supplementary Material; further inquiries can be directed to the corresponding author.
